# Impact of graphene-incorporated nanofillers on material properties and performance of polymers used for prosthodontic patients: a systematic review and meta-analysis

**DOI:** 10.1186/s12903-025-06867-6

**Published:** 2025-09-23

**Authors:** Lamis Ahmed Hussein, Mohamed R. Mahmoud, Mostafa Omran Hussein, Mohammad Rayyan, Ahmed Naguib, Mohamed Sayed

**Affiliations:** 1 Department of Dental Biomaterials, Faculty of Dentistry, Misr International University, Cairo, Egypt; 2 General Dentistry Department, Eastman Institute for Oral Health, University of Rochester, Rochester, NY USA; 3 Department of Removable Prosthodontics, College of Dentistry, Ahram Canadian University, 6th of October, Egypt; 4 Department of Fixed Prosthodontics, Faculty of oral and dental medicine, Misr University for Science & Technology, 6th of October, Egypt; 5 Department of Fixed Prosthodontics, Faculty of Oral and Dental Medicine, Cairo University, Cairo, Egypt; 6 Professor, Intern Department, Future University, Cairo, Egypt; 7 Department of Restorative Dental sciences, Fixed Prosthodontics, Faculty of Dentistry, Ahram Canadian University, 6th of October, Egypt

**Keywords:** Dental prosthesis, Graphene, Graphene oxide, Meta-analysis, Prosthodontics, Polymers

## Abstract

**Background:**

Graphene has been successfully used for years in several applications. It had great potential for use in the medical field. Recently, incorporating graphene into dental biomaterials manufacturing has revolutionized prosthodontics. There is no clear data about the benefit and concentration of adding adding graphene or its derivatives into polymers used in prosthodontics. Accordingly, this review aimed to test the hypothesis that incorporating graphene or its derivatives into prosthodontic polymers, even at low concentrations, improves mechanical properties compared to conventional materials, without compromising biocompatibility.

**Methods:**

The databases (PubMed, Web of Science, Cochrane Library and Willey) were searched based on the designed search strategy. The search strategy was developed using accessible terms, such as, ‘graphene,’ ‘prosthodontic polymers,’ ‘PMMA,’ ‘mechanical properties,’ and ‘dental nanocomposites.’ including medical search topic terms (MESH) where possible, in line with the PICO question. The search process did not include any filters for date or language. Quality assessments were undertaken in the included articles. Meta-analysis was calculated using the standardized mean difference as an effect size, considering the random effect model. Cochran Q and inconsistency I^2^ tests were applied for testing heterogeneity. Based on nanofillers’ concentration, subgroup analysis was applied and interpreted as forest plots. Sensitivity and publication bias testing were also considered before interpretation.

**Results:**

Out of 756 records from databases and other sources, only 22 articles were considered for data extraction according to the inclusion criteria. Eighteen studies were valid for meta-analysis, including seven material properties: flexural strength, impact strength, compressive strength, Shore D hardness, Shore A hardness, Vicker’s hardness and surface roughness. Subgroup analysis, which relied on nanofiller concentration, showed enhanced outcomes at low concentrations. Low sensitivity and variable publication bias levels were recognized.

**Conclusions:**

While low-concentration graphene shows optimized prosthodontic nanocomposites’ properties, high heterogeneity and methodological variability across studies preclude definitive clinical recommendations. Future research must prioritize standardized protocols and clinical validation.

**Trial registration:**

PROSPERO CRD42024522295.

## Background

**Fig. 1:**
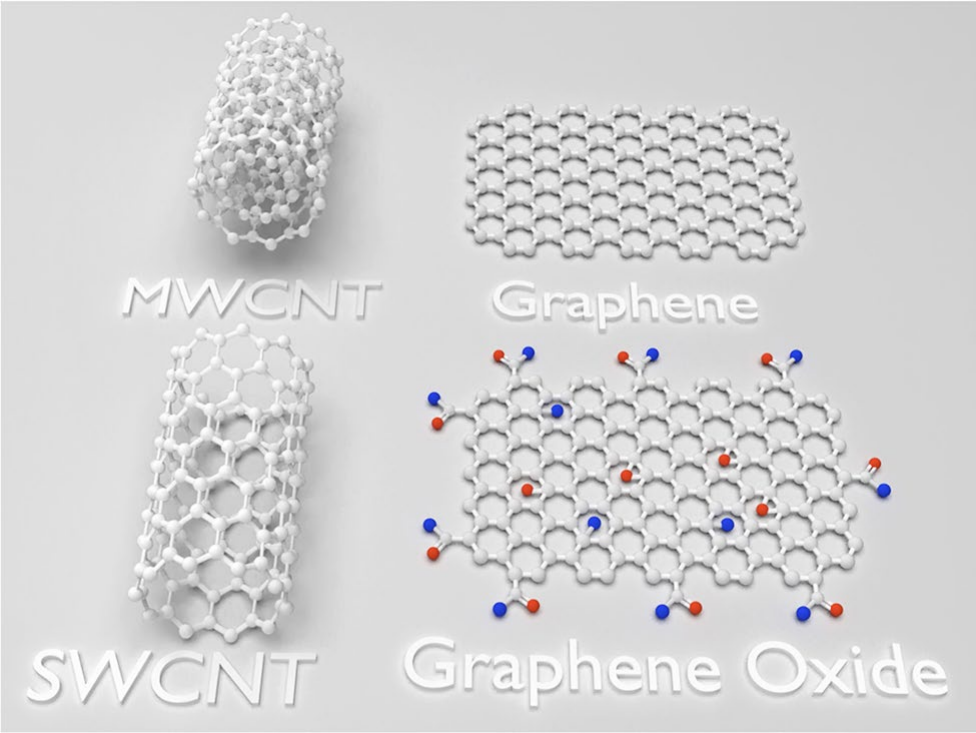
Graphical presentation at a molecular level showing graphene honeycomb structure, graphene oxide, single-walled carbon nanotubule (SWCNT) and multi-walled carbon nanotubule (MWCNT). The white-colored atom is carbon, the red-colored is oxygen and the blue is hydroxide group

Bonding between carbon atoms produces a variety of allotropic forms of carbon with distinct physical properties. The known artificial allotropes are fullerenes, nanotubes (single or multi-walled) and graphene. Graphene nanomaterial is a two-dimensional monolayer of carbon atoms arranged in a hexagonal honeycomb lattice (Fig. 1) [1–3]. Its carbon allotropic structure possesses extraordinary mechanical properties, a high surface area, and good electrical and thermal conductivity [4–10]. It is also characterized by biocompatibility and antimicrobial action [11–15]. 

There are several forms of graphene, encompassing graphene oxide (GO), its reduced form (rGO), graphene nano-platelets (GNPs), and pristine graphene (Fig. 1) [16–19]. Each type of graphene offers unique characteristics and can be modified to satisfy specific needs in dental prosthetics. Pristine graphene provides excellent mechanical reinforcement, while GO and rGO exhibit enhanced dispersibility and compatibility with polymer matrices [16, 20]. Moreover, GNPs compromise mechanical reinforcement and ease of processing [21]. 

Graphene and its derivatives have been used for years in several applications. It had immense potential for use in the medical field [1–6, 22]. There are various key areas where graphene could be beneficial. Graphene-nanocomposites could be used as efficient drug-delivery vehicles and biomedical imaging techniques, such as magnetic resonance imaging (MRI) contrast enhancement and fluorescence imaging [8, 22–25]. Graphene-based biosensors have enhanced selectivity and sensitivity to detect biomolecules, microbes, and pathological markers [26]. Graphene compounds are also performed efficiently in tissue engineering, wound healing, therapeutics, neural interfaces and applications [27–30]. 

The graphene nanofiller’s antimicrobial effect renders it a good candidate for dental products such as mouthwashes, toothpaste and dental implants [31, 32]. Studies on graphene have shown that it reduces oral infections, periodontal disease risk and implantitis. In addition, scaffolds made of graphene have the potential to encourage the regeneration of periodontal tissues by offering a matrix that facilitates cell adhesion, proliferation, and differentiation [33–38]. Applying graphene coatings to the implant-tissue interface can improve biocompatibility and long-term osseointegration [39, 40]. Graphene-based microcarriers can target and regulate medication administration in oral healthcare applications [41–44]. 

Recently, incorporating graphene into dental biomaterials manufacturing has revolutionized prosthodontics. Graphene nanocomposites were tested for denture base, soft liner, single and multi-unit fixed partial dentures, telescopic dentures and removable partial denture esthetic clasps [31, 45–50]. Several manufacturing modes were considered, such as subtractive and additive prototyping and compression molding [46, 51–55]. They have assessed mechanical properties, such as flexural strength, elastic modulus, cyclic fatigue, impact, elongation, wear, roughness and microhardness [4, 6, 8, 10, 12, 45, 47, 50, 51, 53, 56–60]. In addition, physical properties were also studied, including water contact angle, glass transition temperature (Tg), translucency, water sorption and solubility [5, 11, 13, 48, 49, 60]. They reported different biological characteristics using cytotoxicity tests, antimicrobial activity and adhesion [21, 22, 53, 55]. 

The graphene concentration within the polymer matrix significantly influences dental prosthetics’ material properties and performance. Moderate concentrations of graphene increase the nanocomposite materials’ mechanical strength, modulus, and wear resistance [52, 56–58, 61, 62]. However, excessively high concentrations may compromise the material’s properties, processability and biocompatibility. It was also challenging to have acceptable optical properties while keeping higher concentrations [51, 54, 56, 57, 60, 63]. Some studies have highlighted the deterioration in the flexural strength by increasing the graphene’s filler concentrations [51, 52, 55]. They showed the same Hardness and impact strength deterioration, especially in higher concentrations (0.5 and 0.6% per weight) [49, 51, 52]. Therefore, optimizing the graphene concntration is crucial to achieving the desired balance between mechanical enhancement and clinical suitability [31, 51]. Most of the studies compared the incorporation of graphene in different forms and combinations with other nanofillers in concentrations between (0.005 to 1) percent per weight [31, 49, 53, 63]. 

Based on a comprehensive literature search, no in-depth study with quantitative data synthesis was revealed to fill our knowledge gap about the impact of adding graphene at different concentrations into prosthodontic polymers. Accordingly, the objective of this review was testing the hypothesis that incorporating graphene or its derivatives into prosthodontic polymers even at low concentrations may improve mechanical properties compared to conventional materials, without compromising biocompatibility.

## Methods

### Eligibility criteria and protocol registration

This systematic review was designed following the updated “Preferred Reporting Items for Systematic Reviews and Meta-Analyses” (PRISMA) statement [64]. The review was planned to answer the population, intervention, comparator and outcome (PICO) question” In patients requiring dental prosthetics, does the incorporation of graphene or its derivatives into prosthodontic materials compared to other prosthodontic polymeric materials without graphene result in improved material properties and performance? “. Consequently, the protocol was written after a discussion panel among authors to get a complete consensus. Finally, the review protocol was registered in the “international database of prospectively registered systematic reviews” (PROSPERO) under registration No (CRD42024522295).

### Information sources

Four databases were selected to retrieve articles: PubMed, Web of Science, Cochrane Library and Wiley. Other sources were added later, following the snowballing of the eligible records and manual search techniques. No further search processes were conducted after 15 Feb 2024.

### Search strategy

According to the previous PICO question, the search strategy was formulated using the suitable available terms with and without wildcards, including the medical search subjects terms (MESH) whenever possible. A search terms’ boolean process was applied using (OR, AND, NOT) terms as needed. A blank outcome field was set to collect any properties studied in the literature. Both the inclusion and exclusion criteria were set to match the search strategy and support the PICO question. No date or language limits were set as filters during the search process. All search terms’ boolean process, inclusion and exclusion criteria, and distribution on the PICO framework and databases can be seen in (Tables 1 and 2).

**Table 1:** Search strategy details associated with the number of records detected in each database

			PubMed	WoS	CoCh	WL
P: In patients requiring dental prosthetics	**#1**	((((((((“Dentures” [Mesh]) OR (“Dental Prosthesis, Implant-Supported” [Mesh])) OR (Implant-retained)) OR (Implant-assisted)) OR (“Mouth, Edentulous” [Mesh])) OR (Denture*)) OR (Prosthesis)) OR (“Prosthodontics” [Mesh])) OR (Prostheses)	683,186	50,414	20,327	40,322
I: Does the incorporation of graphene or its derivatives into prosthodontic materials	**#2**	((((((((“Graphite” [MeSH Terms]) OR (“graphene oxide” [Supplementary Concept])) OR (“graphene oxide”)) OR (graphene*)) OR (graphen*)) OR (“reduced graphene oxide”)) OR (“silver graphene” [Supplementary Concept])) OR (“GO”)) OR (“rGO”)	246,928	770,187	16,944	876,521
C: Compared to other prosthodontic polymeric materials without graphene	**#3** OR	((((((((“polymethyl methacrylate” [MeSH Terms]) OR (“polymethyl methacrylate”)) OR (polymethyl-methacrylate*)) OR (PMMA)) OR (Acrylic*)) OR (Acrylate*)) OR (“Acrylic resin”)) OR (Resin*)	171,118	401,022	9525	32,204
	**#4**	(((((((“polyetheretherketone” [Supplementary Concept]) OR (“PEEK”)) OR (“Polyaryletherketone”)) OR (PAEK)) OR (“polyacetal” [Supplementary Concept])) OR (polyamide)) OR (nylon)) OR ((“Polymers“[Mesh]))	990,031	2,456,818	24,368	2,134,520
	**#5**	#3 OR #4	1,068,747	2,681,217	28,894	2,078,921
O: Result in improved material properties and performance						
	Search results of ((#1) AND (#2)) AND (#5)		565	93	21	92

**Table 2:** Inclusion and exclusion criteria assigned based on study design and PICO scope domains

	Inclusion criteria	Exclusion criteria
**Study design**	Randomized controlled trials (RCTs), non-randomized controlled trials, prospective cohorts, retrospective cohort, case-control, cross-sectional, and in-vitro studies were included.	Letters to the editor, case reports, case series, reviews, opinion articles, and conference abstracts were excluded.
**Population**	Patients of any age require dental prosthetics, including but not limited to crowns, fixed and removable dentures, and implant prosthetics. Animal models or in-vitro experiments related to dental prosthetics were also considered.	Studies investigated the use of graphene in non-prosthodontic dental materials or applications. Studies where graphene was used in combination with other materials and the effects of graphene cannot be isolated.
**Intervention**	Studies investigating the incorporation of graphene or its derivatives into prosthodontic materials were included.	Studies investigated the use of graphene in non-prosthodontic dental materials or applications. Studies were excluded when graphene was combined with other materials, and its effect could not be isolated.
**Comparator**	Studies compared graphene-incorporated prosthodontic materials to traditional or other polymeric materials without graphene.	Studies compared different formulations of graphene-incorporated materials without a comparison to traditional prosthodontic materials. Studies compared graphene-incorporated materials to non-polymeric dental materials (e.g., metals, ceramics) without comparison to traditional or other polymeric materials.
**Outcome Measures**	Mechanical material properties such as compressive strength, flexural strength, wear resistance, Hardness, impact strength and fracture toughness were included. Physical and biological performance outcomes such as biocompatibility, antimicrobial, longevity, stability, and resistance to degradation were included.	Studies did not report relevant outcomes related to material properties, performance, or adverse effects. Studies reported outcomes related to the fabrication process or cost-effectiveness without relevance to material properties or performance improvement.

### Study selection and data collection

All records were exported from the databases and other sources in endnote format, where all duplicate records were detected and removed in the endnote citation manager software. The resultant citation records were then imported to the screening website (QCRI; rayyan.qcri.org) to facilitate the blind screening process. Two authors (L.H & M.M) were involved independently in the screening process, where the title and abstract were checked against the inclusion and exclusion criteria. Any conflict was resolved after meeting the reviewers and the review moderator (M.R). The decision for eligibility was supported after the full article screening step.

A data extraction sheet was created to collect data from the eligible articles. Items for extraction were designed to comprise both input and output data. The extracted data focused on the type of study groups, range of graphene concentration, structure and manufacturing process. In addition, it included types and test names of the properties studied and conclusions drawn from the studies.

### Study risk of bias assessment

The quality of the collected articles was assessed for risk of bias using the appropriate tool that matched the study design type. For example, the risk of bias tool from Cochrane (ROB II) was proposed for RCTs and Newcastle-Ottawa Scale (NOS) for non-randomized studies. QUIN tool was proposed for the animal models [65], RoBDMAT for dental materials laboratory studies [66] and ROBFEAD for finite element studies [67]. Two investigators (L.H & M.M) checked the risk of bias independently and the conflict points were resolved in a meeting with the moderator. The inter-investigator agreement was checked using the Kappa (K) test to analyze assessment reliability. All quality assessment data were collected in an Excel sheet containing the assessment domain following each tool used.

### Effect measure and data synthesis

The articles included were checked for validity in the quantitative data synthesis. If applicable, the quantitative data extracted from eligible articles were revised to select the appropriate effect size. The standardized mean difference (SMD) was considered for continuous data. As a result, the data, including means, standard deviations, and sample sizes, were extracted from both the control and experimental groups. If data is missing from any article, the authors were contacted by email to be fulfilled. A follow-up email was proposed to be sent four days after the first email. If no response was received after the follow-up email, the study was omitted from the data synthesis step.

After the data for quantitative synthesis became ready, the comprehensive meta-analysis software (Comprehensive Meta-analysis, version 3.3, Biostat, Englewood, NJ, USA, 2005) was used to analyze the data statistically. Upon demand, the meta-analysis considered fixed and random effects models, confirmed with heterogeneity test analysis. Cochran Q and inconsistency I^2^ tests were applied to check and measure heterogeneity, respectively. The (I^2^) test value above 75% is considered high heterogeneity, 50% moderate, while less than 25% low heterogeneity between-study. Forest plots, including studies’ weights, of each outcome were generated after calculating effect size (E.S.), standard errors (S.E.) and confidence intervals (C.I.s) at 95% level where *P* < 0.05 was considered significant.

### Risk of publication bias and additional analyses

A subgroup analysis was proposed to minimize heterogeneity based on the potential factors detected. In order to check the sensitivity of the results of the studied outcomes, the (one study removed) method was applied and presented as forest plots. The publication bias, if applicable, was assessed by Funnel’s plots and Egger’s tests (at *P* < 0.05).

## Results

### Study selection and characteristics

**Fig. 2:**
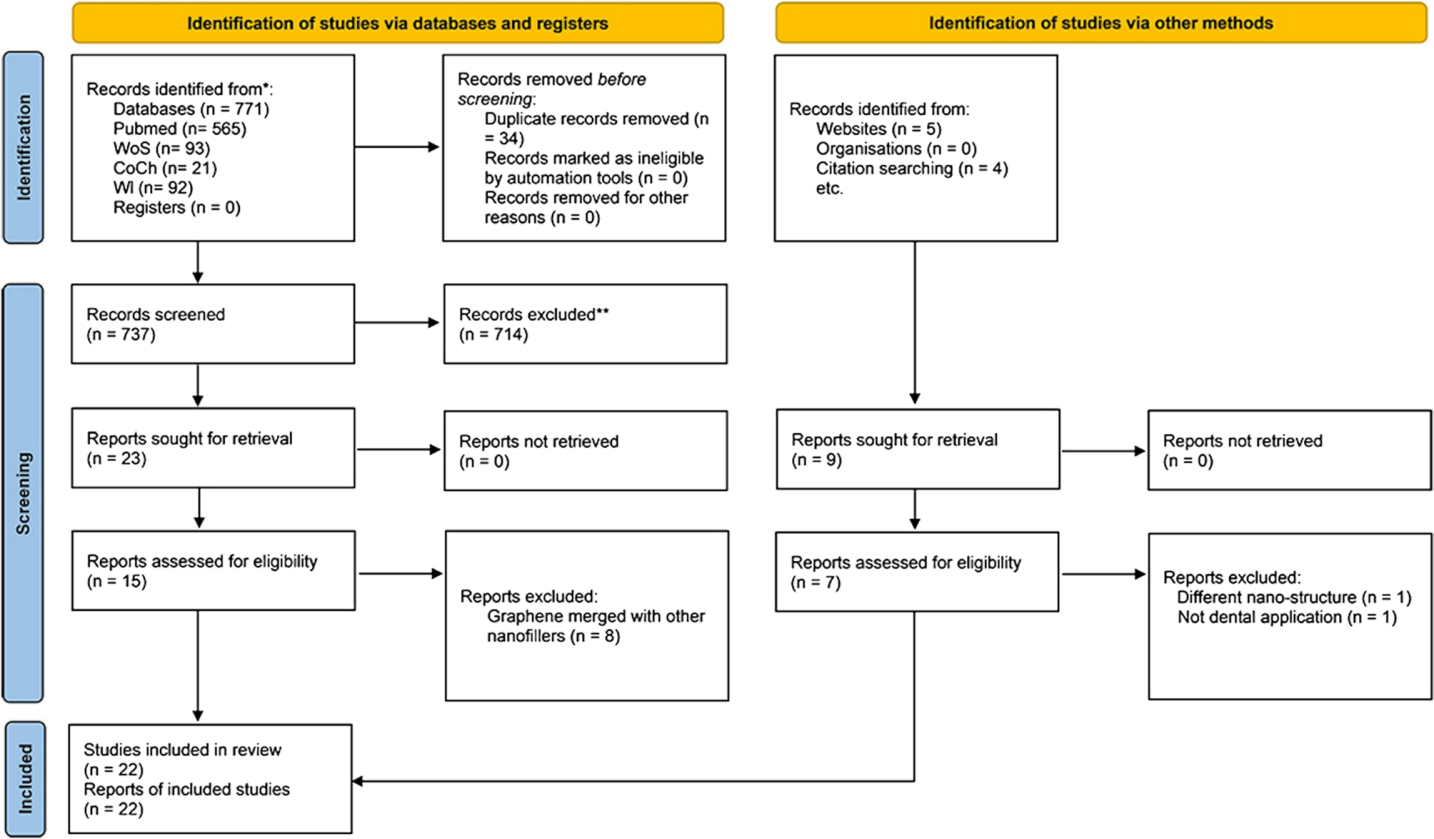
PRISMA flow chart showed details of records included and excluded in different screening phases

The PRISMA flow chart reported all details about the source, the number of records included and the causes of exclusion (Fig. 2).

The total number of electronic records identified from the databases was (771) distributed as PubMed (*n* = 565), WoS (*n* = 93), CoCh (*n* = 21) and Wl (*n* = 92). After deduplication, 737 were screened by title and abstract, where only twenty-three reports were sought for retrieval for full-text assessment (Table 1; Fig. 2). In the full-text exclusion step, only (*n* = 15) primarily involved studies, many studies were excluded where graphene effects could not be isolated (e.g., hybrid nanofillers) or non-prosthodontic applications were tested (e.g., orthodontic brackets). Similarly, nine reports were identified from other sources (websites *n* = 5, citation search *n* = 4). Eight out of fifteen studies were excluded from the database-identified section due to difficulty separating the effect of the studied nanofiller from other nanofillers in the study. On the other hand, two studies were excluded from the other sources section; one was marked as a different nano-structure configuration and one was not for dental application. Finally, twenty-two studies were included in the review analysis.

The included studies were original articles published between 2018 and 2023. They comprised twenty invitro studies, one in-silico (FEA) [50] and one used both invitro and in-silico [47]. Only two in vitro studies performed antimicrobial activities and adhesion tests besides mechanical or physical testing [21, 63]. Polymethyl Methacrylates (PMMA) were represented intensively in the studies as a control group (20 times), while PEEK was mentioned only two times [47, 50]. Four forms of graphene were mentioned: graphene nano-fibers (GNF), graphene nano-platelets (GNP) and nano-graphene oxide sheets (nGO sheets). Graphene concentration ranged between (0.0025 and 2% per weight), added tothe monomer or polymer or both. Samples were manufactured either by compression molding, CAM or 3D printing. Several properties, such as mechanical, physical, and biological properties, were tested. Popular tests were frequently applied to test each property; for example, 3-point bending was used for flexural strength, Vickers test for Hardness, and Izod test for impact strength.

Fatigue was reported in one article using a chewing simulator [45]. Similarly, one study assessed the translucency of the specimens using a Colorimeter. Physical properties encompassing water sorption, solubility and glass transition temperature were mentioned in three studies [22, 48, 49]. More details can be seen in (Table 3). It should be mentioned that graphene was not included in the control group in nearly all of the studies.

**Table 3:** Study characteristics and information showing each study design, groups, sample size, graphene type, concentration and sample fabrication method

Author & year	Study Design	Study groups	Sample size	Graphene Type	Graphene Concentration	Fabrication Methods	Material Properties	Testing methods	Conclusions
Swaroop et al., 2023 [51]	In vitro	PMMA no nanofillers - CG; 0.5% by weight of G; 0.5% by weight of MWCNT; 0.25% by weight of both groups I added to the polymer group II in Monomer	80 specimens, 5 samples per each	GNF	0.5 wt% total one type or mixed 1:1	Compression molding	Flexural strength, impact	Three-point bending & Izod	Nanofillers are better to be added to the monomer of heat-cure PMMA; 0.5% by weight of MWCNT has shown the highest F.S. and I.S. when added to the monomer.
Salgado et al., 2022 [21]	In Vitro	PMMA no nanofillers - CG; 0.01% by weight of GNP; 0.1% by weight of GNP; 0.25% by weight of GNP; 0.5% by weight of GNP	70 specimens (cylinder), 14 samples per each 25 specimens (bar), 5 samples per each	GNP	0.01–0.5 wt%	3D printing	Antimicrobial activity and adhesion, Surface roughness	Microbial Growth Inhibition Assay Microbial Adhesion Assay contact profilometer	Graphene increases surface roughness and inhibits C. albicans and S. mutans growth and adhesion.
Swami et al., 2018 [56]	In Vitro	PMMA no nanofillers - CG; 0.5% by weight of G; 0.5% by weight of MWCNT; 0.25% by weight of both	80 specimens, 10 samples per each	GNF	0.5 wt% total one type or mixed 1:1	Compression molding	Flexural strength, impact	Three-point bending & Izod	Adding 0.5% by Wt of (MWCNT) showed the highest flexural and impact strength between groups
Selva-Otaolaurruchi et al., 2023 [45]	In Vitro	(PMMA group) without graphene and (PMMA-G group) doped with graphene.	44 specimens 22 per group	GNF	0.15–0.175% PPM	CAD CAM	Mechanical fatigue	Cyclic loading using a chewing simulator until fracture or 240,000 load applications	Graphene-doped PMMA restorations exhibit higher mechanical fatigue resistance than machined PMMA restorations, suggesting their potential as a new restorative material in implant prosthetics.
Salgado et al., 2023 [52]	In Vitro	PMMA no nanofillers - CG; 0.01% by weight of GNP; 0.1% by weight of GNP; 0.25% by weight of GNP; 0.5% by weight of GNP	15 bar-shaped (80 × 10 × 4 mm) specimens 25 round-shaped 5 per group 25 bar-shaped (50 × 10 × 4 mm) 5 per group	GNP	0.01–0.5% by weight	3D printing	Flexural strength, hardness and surface roughness	3-point bending Shore D contact profilometer	Graphene seems to be viable at low concentrations to improve mechanical properties without prejudice to the surface roughness of a 3D-printed PMMA.
Punset et al., 2022 [31]	In Vitro	PMMA no nanoillers - CG; 0.027% by weight of nGO	15 specimens for Hardness 5 per group in others	nGO sheets	0.027% by weight	CAD CAM	Hardness, compression test, and surface wear resistance	Shore D, Vickers hardness, fracture toughness Pin-on-Disc Test	Adding as low as 0.027% by weight of graphene nanosheets to PMMA can improve the mechanical properties. More studies are needed to add more concentration without jeopardizing the esthetics
Ortensi et al., 2024 [46]	In Vitro	PMMA no nanoillers - CG; 0.027% by weight of nGO	60 specimens (3-unit bridge), 10 per group	nGO sheets	0.027% by weight	CAD CAM	Fracture strength and linear elongation at break directly and after Thermal (30,000 cycles) & Mechanical (6550 cycles) aging	Fracture load test	Graphene nanomaterials can improve mechanical properties (fracture strength and elongation at break) and are not affected by aging.
Lee et al., 2018 [63]	In Vitro	PMMA no nanoillers - CG; 0.25% by weight nGO 0.5% by weight nGO 1% by weight nGO 2% by weight nGO	10 per group for F.S. 5 per group for all others	nGO sheets	0.25-2% by weight	Compression molding	Flexural strength, Hardness, Antimicrobial adhesive effects	3-point bend test, Vickers hardness non-thermal oxygen plasma treatment	Incorporating nGO showed anti-adhesive effects against microbes, which lasted after 28 days.
Khan et al., 2022 [49]	In Vitro	PMMA no nanoillers - CG; 0.1% by weight of nGO 0.3% by weight of nGO 0.6% by weight of nGO	60 specimens 15 per group	nGO sheets	0.1–0.6% by weight	Compression molding	Surface roughness, water contact angle, Hardness, water sorption and solubility	Profilometer, tensiometer device, Shore A durometer, weigting method	nGO sheets minimized the Hardness of RDL, along with good surface roughness and wettability properties. Less water sorption and solubility were observed than in the control group.
Khalil & Enaba 2021 [53]	In Vitro	PMMA no nanoillers - CG; 0.02% by weight nGO 1% by weight TiO_2_ 1% by weightTiO_2_/0.02% nGO 0.02% by weight GOCUR 0.02% TiO_2_/0.02% GOCUR	78 specimens 7 per group for F.S. 6 per group or H	nGO sheets	0.01–0.02% by weight	Compression molding	Flexural strength, Hardness	3-point bending test Vicker hardness	Using nGO, TiO2 and GOCUR nanofillers individually or in combination significantly increases the flexural strength and Hardness of PMMA material.
Ionescu et al., 2022 [22]	In Vitro	PMMA no nanoillers - CG; < 50 ppm GNF	20 specimens *n* = 10 (UTS) 48 specimens *n* = 24 (FS) 20 specimens *n* = 10 (W.S., SL) 6 specimens *n* = 3 (HPLC)	GNF	< 50 ppm	CAD CAM	UTS, flexural strength, water sorption, solubility, HPLC	Tension test, 3-point bending, weighting method, HPLC	Adding graphene to PMMA improved the material’s ultimate tensile strength (UTS) and flexural strength. The water sorption (W.S.) and solubility were found to be decreased. (HPLC) also showed a tiny amount of elution.
Hussein 2022a [47]	In Vitro & In silico	PEEK no nanoillers; 0.027% by weight GNF	32 specimens 16 per group	nGO sheets	0.027% by weight	CAD CAM	Cyclic retention force, Mechanical deformation, Maximum principal stress and strain	Cyclic insertion and removal (10^−4^ cycles) with thermocycling, Digital 3D deviation markers, FEA	nGO showed less performance related to retention force, deformation, and stress-strain concentrations than PEEK. Stress and strain concentration zones were similar, which seems to be a common location in clasp failure.
Hussein 2022b [50]	In silico	PEEK no nanoillers; 0.027% by weight GNF	NA	nGO sheets	0.027% by weight	CAD CAM	Von Mises stress and deformation	FEA	PEEK showed better stress distribution and low deformation as RPD telescopic framework material than nGO PMMA.
Ghosh & Shilpa 2020 [57]	In Vitro	PMMA no nanoillers - CG; 0.5% by weight of MWCNT; 0.5% by weight of nGO	60 specimens 20 per group	nGO sheets	0.5% by weight	Compression molding	Flexural strength	3-point bending test	0.5 wt% MWCNT is an effective additive for PMMA to increase the flexural strength compared to 0.5 wt% graphene oxide.
Di Carlo et al., 2020 [58]	In Vitro	PMMA no nanoillers - CG; 0.027% by weight of nGO	40 specimens 20 per group	nGO sheets	0.027% by weight	CAD CAM	Flexural strength	3-point bending test	The use of graphene nanofillers enhances PMMA strength. It is recommended that the investigation be extended by testing fatigue properties.
De Angelis et al., 2023 [61]	In Vitro	PMMA no nanoillers - CG; 0.027% by weight of nGO BACR	30 specimens *n* = 10 (FS) 30 specimens *n* = 10 (CS) 30 specimens *n* = 10 (VH)	nGO sheets	0.027% by weight	CAD CAM	Flexural strength, Compressive strength, Hardness	3-point bending test compressive test Vickers hardness	The addition of Graphene to PMMA showed inferior mechanical properties compared to conventional PMMA and BACR
Ciocan et al., 2021 [62]	In Vitro	Three PMMA no nanofillers Multilayered; 0.027% by weight of nGO One Hybrid ceramic Two organically modified ceramics	21 specimens 3 per group	nGO sheets	0.027% by weight	CAD CAM	Micro-mechanical properties (Continuous Contact Stiffness, Micro-Elastic Modulus and Micro-Hardness)	Nano-indentation	Hybrid ceramic-based CAD/CAM showed the best micro-mechanical properties, followed by the CAD/CAM PMMA-graphene, with a high level of homogeneity compared to other materials. The multilayered PMMA had the lowest value of the tested materials.
Çakmak et al., 2023 [59]	In Vitro	PMMA no nanoillers (CAM & 3D printed) - CG; 0.027% by weight of nGO	60 specimens 20 per group (with and without thermocycling)	nGO sheets	0.027% by weight	CAD CAM & 3D printed	Flexural strength, Micro-hardness	3-point bending test Vickers hardness	Graphene-PMMA CAD-CAM discs showed the highest flexural strength even after thermocycling. However, it also showed the highest micro-hardness values and the 3D-printed resin did not seem to be affected by thermocycling.
Agarwalla et al., 2019 [60]	In Vitro	PMMA no nanoillers - CG; ≈0.027% by weight of nGO Resin nano ceramic Polymer-infiltrated Lithium disilicate glass-ceramics	60 specimens *n* = 10 (FS) 18 specimens *n* = 3 (VH)	nGO sheets	≈ 0.027% by weight	CAD CAM	Translucency, Hardness, flexural strength and Weibull modulus	Colorimeter, Bi-axial flexural strength, Vickers hardness	Adding graphene-like materials to PMMA showed non-significant effects. Although its effect was similar to that of VE used for single restorations,
Abad-Coronel et al., 2020 [54]	In Vitro	PMMA no nanoillers - CG; ≈0.027% by weight of nGO; Acetal resin; Polysulfone	40 specimens 3 UTB 10 per group	nGO sheets	≈ 0.027% by weight	CAD CAM	Fracture Resistance	Compression Test	Graphene specimens showed the highest fracture stress with less Deformation after Polysulfone.
Aati et al., 2022 [55]	In Vitro	PMMA no nanoillers - CG; 0.025% by weight of GNP; 0.05% by weight of GNP; 0.1% by weight of GNP; 0.25% by weight of GNP	75 specimens *n* = 15 (FS) 75 specimens *n* = 15 (F.T.) *n* = 60 (H) *n* = 6 (CytoT) *n* = 6 (AMA)	GNP	0.025–0.25% by weight	3D printing	Flexural strength, fracture toughness, Micro nano hardness, cytotoxicity, Antimicrobial activity	3-point bending, displacement load after pre-crack, Vickers hardness, nano-indentation, Fibroblast culture incubation, Antibiofilm activity, Biofilm Live/Dead assay	GNPs improved strength at ≤ 0.05 wt%, and up to 0.25 wt% enhanced hardness and elasticity without inducing cytotoxicity. Notably, GNPs showed significant antimicrobial activity, particularly against C. albicans, correlating with filler proportion.
Alamgir et al., 2018 [48]	In Vitro	PMMA no nanoillers - CG; 0.0025% by weight of nGO; 0.0025% by weight of nGO + 1% by weight TiO_2_;	9 specimens *n* = 3	nGO sheets	0.0025% by weight	Compression molding under 270 °C and 300 MPa	X-ray diffraction (XRD), Glass transition temperature (T_g_), Indentation depth and elastic modulus, and scratch analysis	(XRD) analysis, DSC analysis, thermogravimetric analysis (TGA), Multicyclic micro indentation test	nGO group showed less indentation depth than pure PMMA. Young’s modulus of nGO was higher than pure PMMA. It also showed less (T_g_) and (T_m_).

*CG control group, *GNP* graphene nanoplatelets, *GNF* graphene nano fiber, *PPM* part per million, *nGO* nano graphene oxide, *SWCNT* single-walled carbon nanotubes, *MWCNT* multi-walled carbon nanotubes, *nGOCUR* Curcumin Loaded Graphene Oxide, *UTS* ultimate tensile strength, *WS* water sorption, *SL* solubility, *HPLC* High-performance liquid chromatography, *PEEK* poly-ether-ether-ketone, *BACR* bis-acryl composite resin, *3 UTB* 3-unit temporary bridge, *H* hardness, *CytoT* cytotoxicity, *AMA* antimicrobial activity, *FS* Flexural strength, *IS* Impact, *CA* candida Albicans, *SM* S.mutans bacteria, *G-CAM* graphene doped PMMA, *VE* Polymer-infiltrated ceramic-network, *FEA* finite element analysis

### Risk of bias in studies

Invitro laboratory studies assessed by the RoBDMAT tool revealed a risk of bias in some domains. All studies reported the presence of a control group, considering identical experimental conditions, standardizing samples and materials and using adequate and standardized testing procedures/outcomes. In contrast, none of them considered blinding of the testing operator. Randomization of samples was not reported in twelve studies [21, 48, 49, 51–54, 56, 57, 63]. The power of sample analysis was not reported in sixteen studies [21, 22, 31, 52, 54–56, 58–62]and was sufficiently reported in one study [57]. The selection of the appropriate statistical analysis was not reported in three studies [48, 51] and was insufficiently reported in nine studies. Fourteen studies insufficiently reported the correct outcome [21, 22, 45, 46, 48, 51, 53, 56–60, 62, 63]. More details can be seen in (Table 4).

**Table 4:** Risk of bias checklist of the RoBDMAT tool showing the quality assessment status of the laboratory experimental studies in each domain

Author	D1: Bias in Planning and Allocation			D2: Bias in Specimen Preparation		D3: Bias in Outcome Assessment		D4: Bias in Data Treatment and Reporting	
	Control Group	Correct Randomization of Samples	Sample Size Calculation	Identical Experimental Conditions	Standardization of Samples and Materials	Adequate and Standardized Testing Procedures/Outcome	Blinding of the Testing Operator	Appropriate Statistical Analysis	Correct Reporting of Outcomes
Swaroop et al., 2023 [51]	Reported	not reported	not reported	Reported	Reported	Reported	not reported	not reported	insufficiently reported
Swami et al., 2018 [56]	Reported	not reported	Reported	Reported	Reported	Reported	not reported	insufficiently reported	insufficiently reported
Selva-Otaolaurruchi et al., 2023 [45]	Reported	not applicable	Reported	Reported	Reported	Reported	not reported	Reported	insufficiently reported
Salgado et al., 2023 [52]	Reported	not reported	not reported	Reported	Reported	Reported	not reported	insufficiently reported	Reported
Salgado et al., 2022 [21]	Reported	not reported	not reported	Reported	Reported	Reported	not reported	insufficiently reported	insufficiently reported
Punset et al., 2022 [31]	Reported	not applicable	not reported	Reported	Reported	Reported	not reported	insufficiently reported	Reported
Ortensi et al., 2024 [46]	Reported	not applicable	Reported	Reported	Reported	Reported	not reported	Reported	insufficiently reported
Lee et al., 2018 [63]	Reported	not reported	not reported	Reported	Reported	Reported	not reported	insufficiently reported	insufficiently reported
Khan et al., 2022 [49]	Reported	not reported	not reported	Reported	Reported	Reported	not reported	insufficiently reported	Reported
Khalil & Enaba 2021 [53]	Reported	not reported	Reported	Reported	Reported	Reported	not reported	Reported	insufficiently reported
Ionescu et al., 2022 [22]	Reported	not applicable	not reported	Reported	Reported	Reported	not reported	Reported	insufficiently reported
Hussein 2022a [47]	Reported	not applicable	Reported	Reported	Reported	Reported	not reported	Reported	Reported
Ghosh & Shilpa 2020 [57]	Reported	not reported	insufficiently reported	Reported	Reported	Reported	not reported	insufficiently reported	insufficiently reported
De Angelis et al., 2023 [61]	Reported	not applicable	not reported	Reported	Reported	Reported	not reported	Reported	insufficiently reported
Ciocan et al., 2021 [62]	Reported	not applicable	not reported	Reported	Reported	Reported	not reported	insufficiently reported	insufficiently reported
Çakmak et al., 2023 [59]	Reported	not applicable	not reported	Reported	Reported	Reported	not reported	Reported	insufficiently reported
Alamgir et al., 2018 [48]	Reported	not reported	not reported	Reported	Reported	Reported	not reported	not reported	insufficiently reported
Agarwalla et al., 2019 [60]	Reported	not applicable	not reported	Reported	Reported	Reported	not reported	Reported	insufficiently reported
Abad-Coronel et al., 2023 [54]	Reported	not applicable	not reported	Reported	Reported	Reported	not reported	insufficiently reported	Reported
Aati et al., 2022 [55]	Reported	not reported	not reported	Reported	Reported	Reported	not reported	Reported	Reported
Di Carlo et al., 2020 [58]	Reported	not applicable	not reported	Reported	Reported	Reported	not reported	Reported	Reported

In-silico FEA studies were assessed using the ROBFEAD tool. The first study [47] answered (No) in only one question: “Q 11: Were dynamic loading conditions applied? (if applicable)”. Accordingly, it showed an overall low risk of bias. In the second study [50], two questions were answered (No) from the high risk of bias question category “Q 1: Was 3D model developed using DICOM images?” and “Q 11: Were dynamic loading conditions applied? (if applicable)”. These two responses have rendered the study at high risk of bias (Table 5). The reliability of the inter-investigator agreement showed a high level of agreement, ranging from values (0.856 to 1.0).

**Table 5:**
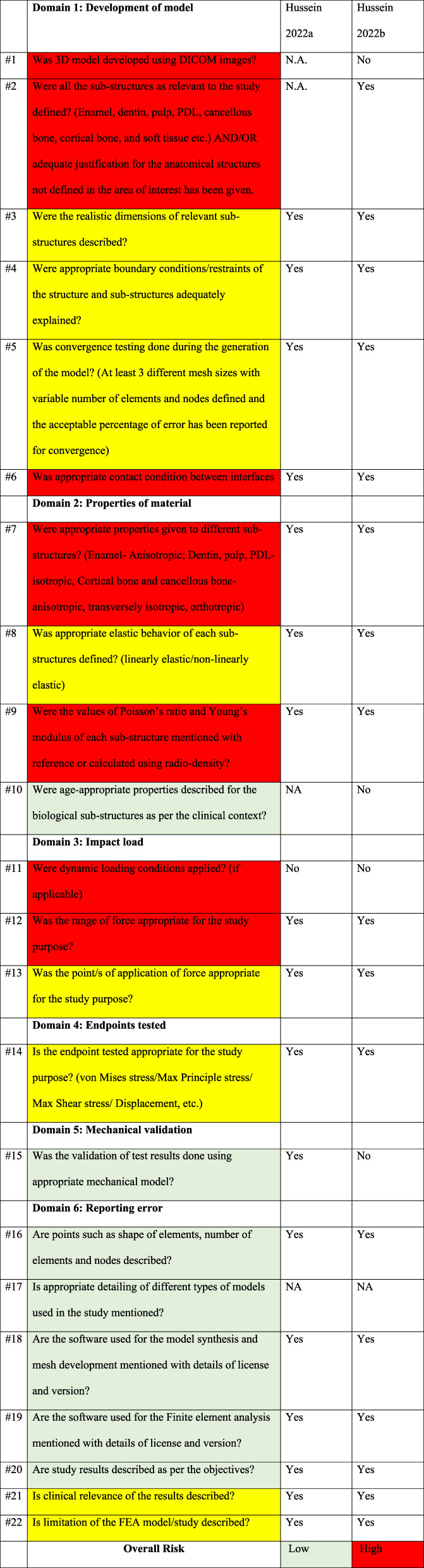
Risk of bias color-coded checklist of the ROBFEAD tool, as stated by its authors [67], showing the quality assessment status of the in-silico finite elements studies in each domain. Red, yellow and green represent high, moderate and low-risk categories

### Data synthesis and nanofillers performance

The impact of incorporating the nanofiller on the studied properties and outcome was reported in (Table 3), where properties studied, testing methods and conclusion of the twenty-two included studies were collected.

Whenever sufficient data were available to provide a valid and meaningful analysis, a series of meta-analyses were applied to eighteen valid studies. Properties included in the meta-analyses encompassed flexural strength in (MPa), compressive strength in (MPa), surface roughness in (µm), Vicker’s hardness number (VHN), Shore A Durometer hardness (S.A. scale), Shore D Durometer hardness (S.D. scale) and impact strength (J/m²). Whenever possible, subgroup analyses were undertaken for each property, considering the nanofillers’ concentration as a parameter.

#### Flexural strength

**Fig. 3:**
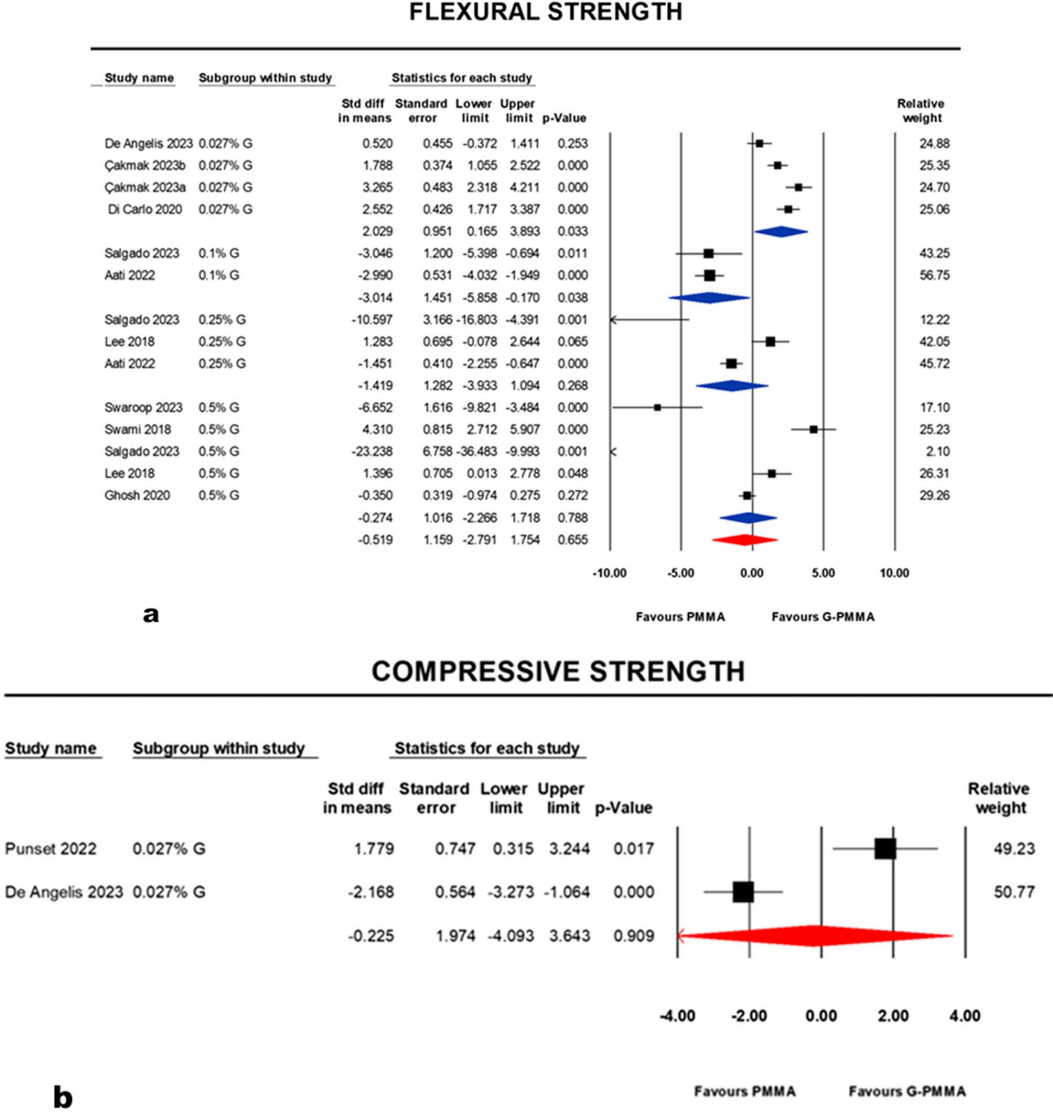
Forest plots and statistics reporting point estimate of standardized mean difference (SMD), standard error (SE), confidence interval (CI), *p*-value and studies’ relative weights in different groups or subgroups. **a**: studies for flexural strength and **b**: studies for compressive strength

Four subgroups within the study of flexural strength were generated. At a concentration of (0.027%), there was a significant difference, favoring the graphene nanofillers group (G-PMMA), was noted: *p* = 0.033 (95% CI: 0.165, 3.893; I^2^ = 84.582%). In contrast, the result at a concentration of (0.1%) was significantly favoring nanofillers-free (PMMA): *p* = 0.038 (95% CI: −5.858, −0.170; I^2^ = 0.00%). At a concentration of (0.25%), no significant difference between PMMA and G-PMMA was noted: *p* = 0.268 (95% CI: −3.933, 1.094; I^2^ = 90.500%). Similarly, at a concentration of (0.5%), no significant difference between PMMA and G-PMMA was noted: *p* = 0.788 (95% CI: −2.266, 1.718; I^2^ = 93.472%). Considering the random effect model, the pooled analysis of the subgroups reported no significant difference between PMMA and G-PMMA: *p* = 0.655 (95% CI: −2.791, 1.754; I^2^ = 94.043%). More details can be seen in the forest plot (Fig. 3, a).

#### Compressive strength

Two studies at a concentration (0.027%) reported significant differences between groups. Punset et al.’s [31] study showed a significant difference in favoring G-PMMA, while De Angelis et al.’s [61] study favored PMMA. Considering the random effect model, the pooled meta-analysis showed an insignificant difference: *p* = 0.909 (95% CI: −4.093, 3.643; I^2^ = 94.380%) (Fig. 3, b).

#### Surface roughness

**Fig. 4:**
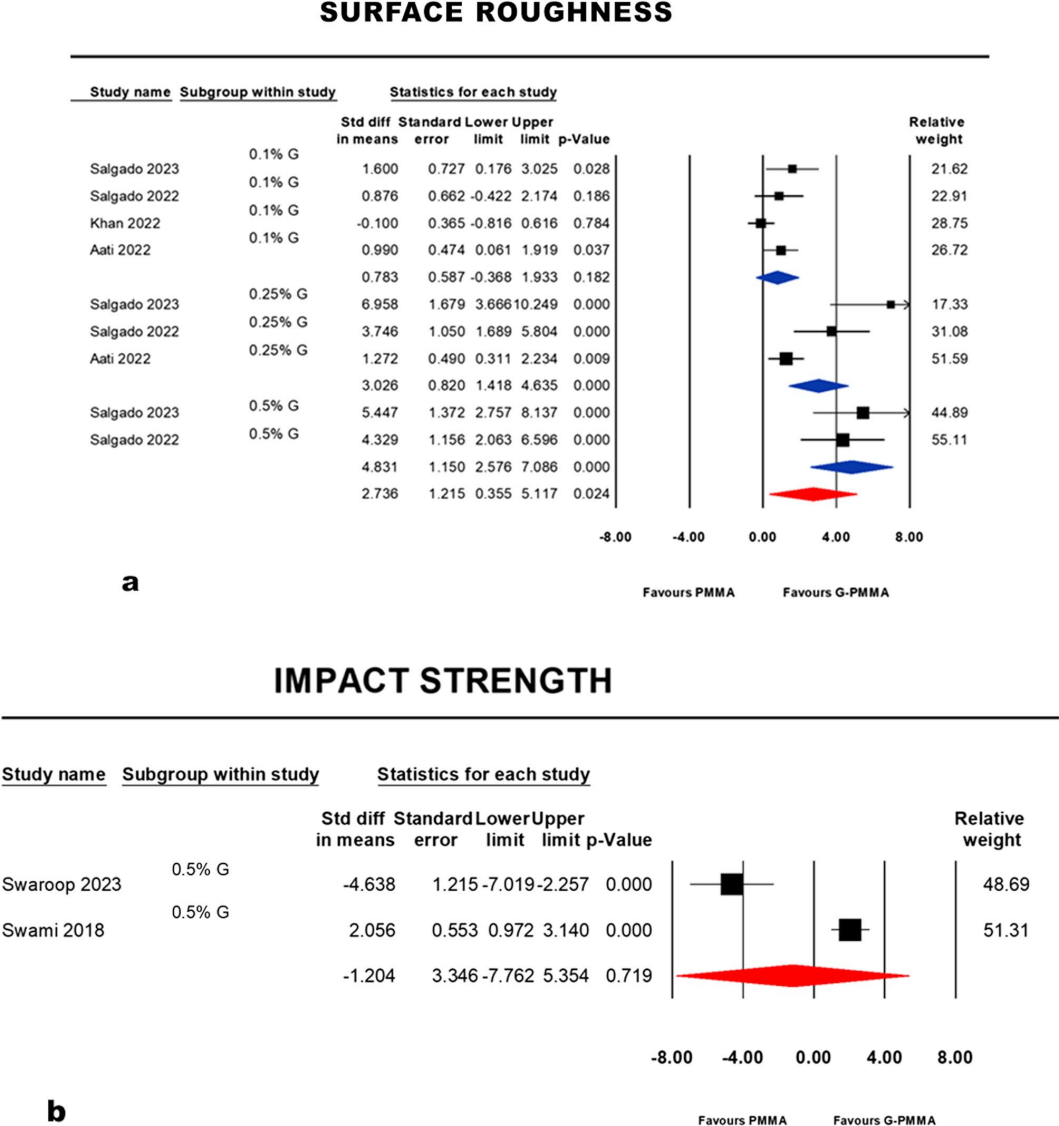
Forest plots and statistics reporting point estimate of standardized mean difference (SMD), standard error (SE), confidence interval (CI), *p*-value and studies’ relative weights in different groups or subgroups of surface roughness (**a**), categorized by concentration parameter. (**b**): the second forest plot for impact strength meta-analysis at a concentration (0.5%)

Three subgroups were formed in the surface roughness meta-analysis. At a concentration of (0.1%), there was no significant difference between PMMA and G-PMMA: *p* = 0.182 (95% CI: −0.368, 1.933; I^2^ = 52.806%). On the contrary, a significant difference favoring G-PMMA at a concentration (0.25%): p = < 0.001% (95% CI: 1.418, 4.635; I^2^ = 85.364%) and at a concentration (0.5%): p = < 0.001% (95% CI: 2.576, 7.086; I^2^ = 0.00%). Considering the random effect model, the pooled meta-analysis showed a significant difference favoring G-PMMA: *p* = 0.024 (95% CI: 0.355 5.117; I^2^ = 83.288%) (Fig. 4, a).

#### Impact strength

The meta-analysis of the impact strength included two studies at a concentration (0.5%). No significant difference was reported as each study favored a different comparison group [51, 56]. Considering the random effect model, the summary effect estimate of the meta-analysis showed an insignificant difference at *p* = 0.719 (95% CI: −7.762, 5.354; I^2^ = 96.025%) (Fig. 4, b).

#### Vicker’s hardness

**Fig. 5:**
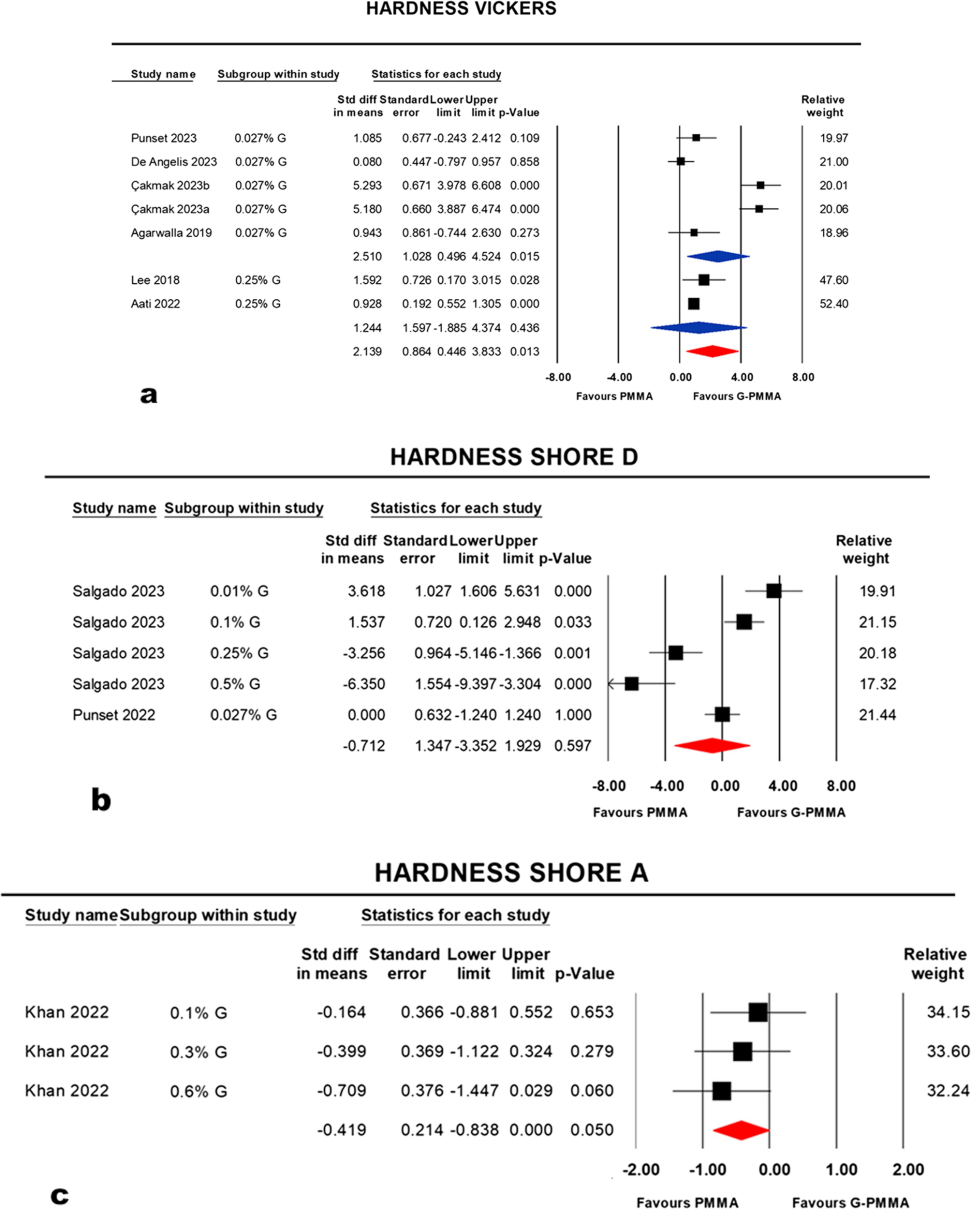
Forest plots and statistics reporting point estimate of standardized mean difference (SMD), standard error (SE), confidence interval (CI), *p*-value and studies’ relative weights in different groups or subgroups of different hardness tests used in the studies. (**a**): Vickers hardness subgroups, (**b**): Shore D hardness test studies and (**c**): Shore A hardness test studies

Two subgroups within the study were reported at (0.027%) and (0.25%) concentrations. At a concentration of (0.027%), there was a significant difference, favoring the graphene nanofillers group (G-PMMA), was noted: *p* = 0.015 (95% CI: 0.496, 4.524; I^2^ = 94.192%). At a concentration of (0.25%), no significant difference between PMMA and G-PMMA was noted: *p* = 0.436 (95% CI: −1.885, 4.374; I^2^ = 0.00%). Considering the random effect model, the pooled estimate of the subgroups showed a significant difference favoring G-PMMA at *p* = 0.013 (95% CI: 0.446, 3.833; I^2^ = 92.644%) (Fig. 5, a).

#### Shore D hardness

Two studies with five concentrations (0.01, 0.027, 0.1, 0.25, 0.5%) were assessed [31, 52]. Using the random effect model, the summary effect estimate of the meta-analysis showed a non-significant difference between PMMA and G-PMMA at *p* = 0.597 (95% CI: −3.352, 1.929; I^2^ = 91.121%) (Fig. 5, b).

#### Shore A hardness

Khan et al., [49] have studied three nanofiller concentrations (0.1, 0.3, 0.6%).Using the random effect model, the summary effect estimate of the meta-analysis showed a significant difference between PMMA and G-PMMA, favoring PMMA: *p* = 0.050 (95% CI: −0.838, < 0.001; I^2^ = 0.00%) (Fig. 5, c).

### Results of sensitivity analyses

**Fig. 6:**
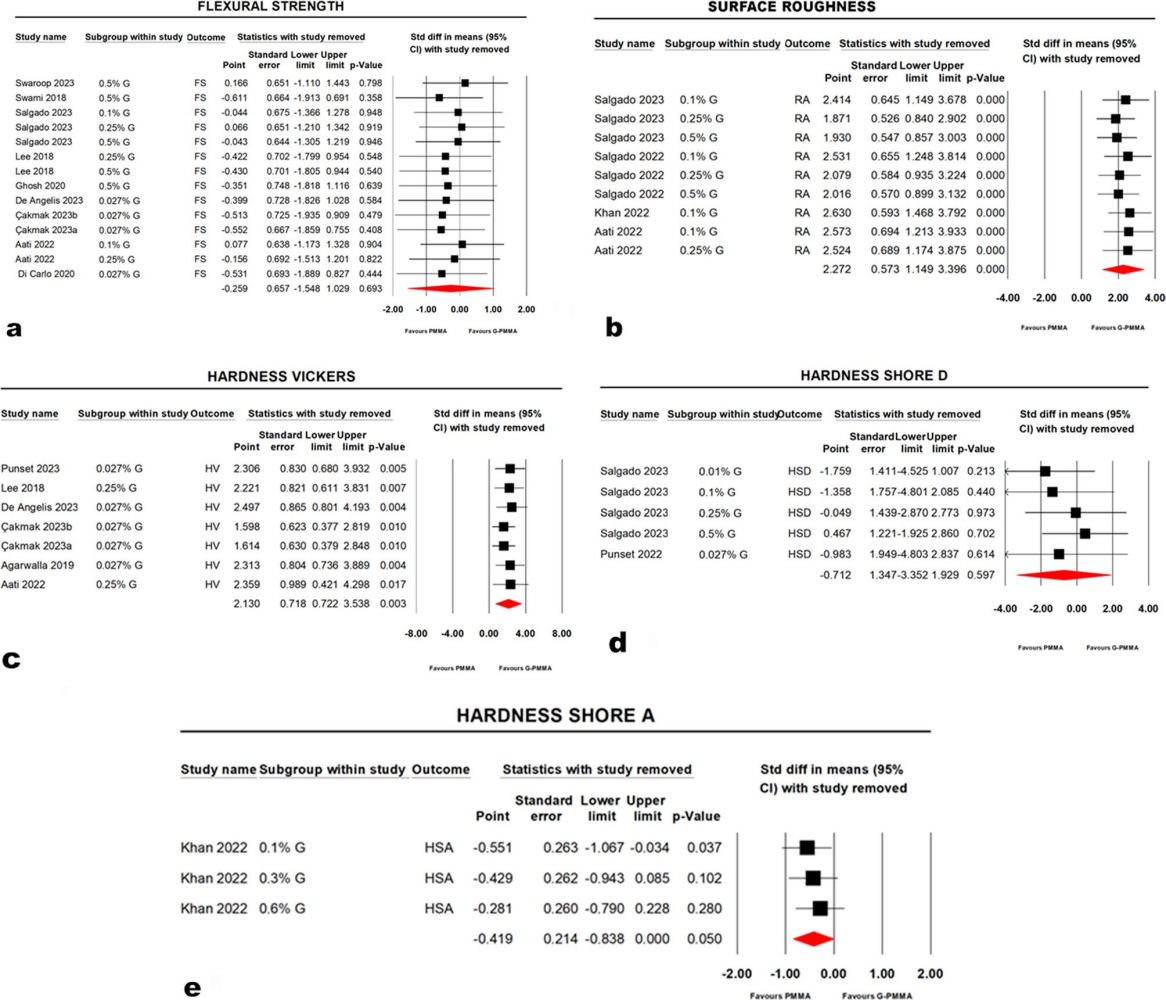
Sensitivity analysis using"one study removed"for different properties. **a**; flexural strength, **b**; surface roughness, **c**; Vickers hardness, **d**; Shore D hardness and **e**; Shore A hardness

A series of one-study-removed meta-analyses were applied to test the results’ sensitivity. The forest plot of the (one study removed) testing the flexural strength showed that three studies seemed to skew the summary estimate of the meta-analysis [51, 52, 55]. However, there was no change in the significance after their removal, with p-values ranging from (0.358 to 0.948) for all (one study removed analysis) (Fig. 6, a). Accordingly, no significant differences throughout the alternatively excluded studies were reported. In the same context, the (CI) widths were shown to be homogenous across analyses, as no marked changes were detected. Similarly, the Shore D forest plot showed the study by Salgado et al., [52] at (0.5%) concentration appeared to shift the summary estimate of the meta-analysis, but without a change in the significance of p-value (*p* = 0.702) (Fig. 6, d).

The inspection of the forest plots of one study removed analysis of surface roughness, Vicker’s hardness, Shore A and impact strength, indicating that it is likely that one study could not significantly influence the summary effect of the meta-analysis or shift the summary estimate in one direction to another (Fig. 6, b, c, e).

### Results of publication bias analysis

**Fig. 7:**
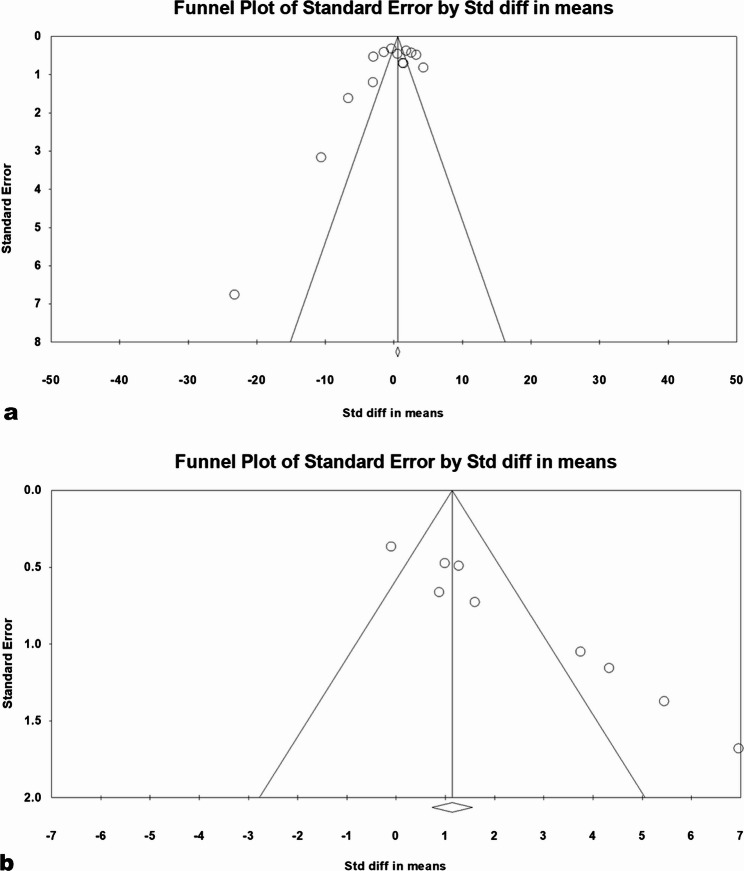
Funnel plots of the flexural strength; **a** and surface roughness; **b** showing standard error by the standard difference in means

Two material properties were illegible for publication bias because of the number of studies available: the flexural strength and the surface roughness (Fig. 7, a and b), respectively. Inspection of the funnel plot of the flexural strength revealed aggregation of the studies in the upper top of the funnel plot on both sides of the estimate’s point, with one study found in the lower left corner [52]. This finding supports the low tendency to publication bias confirmed by the insignificance value of Egger’s test, as seen in Egger’s regression intercept (Table 6).

**Table 6:** Egger’s regression intercept of flexural strength and surface roughness

	Flexural strength	Surface roughness
Intercept	−2.64829	5.12080
Standard error	2.25962	0.54638
95% lower limit (2-tailed	−7.57158	3.82881
95% upper limit (2-tailed)	2.27500	6.41279
t-value	1.17201	9.37218
df	12.00	7.00
P-value (1-tailed)	0.13197	0.00002
P-value (2-tailed)	0.26394	0.00003

In contrast, the surface roughness funnel’s plot showed a scattering of the studies with asymmetrical distribution dominated on the right side. Egger’s regression intercept (Table 6) confirmed the high publication bias tendency where significant differences were recorded at *p* < 0.05.

## Discussion

This study was designed to answer a research question about using graphene or its derivatives to fabricate enhanced nanocomposites suitable for prosthodontic patients. The review focused on prosthodontic polymers and highlighted their mechanical, physical and biological performance. The review was able to quantify the data analysis by a series of meta-analyses for flexural strength, compressive strength, surface roughness, impact strength and Hardness (Figs. 3, 4 and 5). Other properties mentioned in the literature were also described, such as fatigue, water sorption, solubility, thermal, optical and antimicrobial characteristics (Table 3).

Although graphene oxide seems to play a role in modifying the polymers’ properties in many instances, the results generally showed a non-significant effect in the pooled summary of the flexural strength, compressive strength, Shore D and impact strength. Adding graphene oxide as low as (0.027%) per weight (wt) to PMMA polymer showed a significant improvement in the flexural strength of this subgroup (*p* = 0.033). This enhancement deteriorated in higher concentrations, such as (0.1–0.5% wt), where an adverse effect emerged (Figs. 3 and a and 5 and a). These findings coincide with some studies and could be attributed to the nanofiller concentration and their dispersion technique in the polymer matrix, which are the critical elements for the desirable effect [57, 63]. High concentrations are believed to agglomerate and affect the proper distribution of fillers; hence, stress concentration may occur [60]. For such purposes, ultrasonication could be a convenient solution to enhance nanofiller dispersion by vibration and allow homogenization of the particles in the matrix [31]. Another approach was adding nanofillers to the monomer, not the polymer, as Swaroop et al., [51] mentioned. Silanization or chemical modification by the carboxylate group was also suggested [63]. 

In addition, this might happen when the fabrication method complicates incorporating the nanofiller. For example, a study by Aati et al., [55] showed an adverse effect on flexural strength by adding GO to the 3D-printed resin. Conversely, industrial incorporation of GO to PMMA CAD-CAM discs optimized the dispersion process and minimized this issue [31, 59, 61]. 

Hardness is a surface property that represents the resistance to indentation. Significant enhancement was seen in the Hardness measured by the Vickers method (*p* = 0.013). This finding coincides with many studies and could be attributed to the enhancement in polymer bonding in the presence of the nanofiller [10, 59, 63]. However, results of Shore D hardness also showed the same deterioration in Hardness by increasing the concentration from (0.1 to 0.5% wt), leadng to an insignificant pooled summary (*p* = 0.597). In the same context, adding GO, in different concentrations, to the resilient liner was not efficient in enhancing Hardness as tested by the Shore A test [49]. 

Surface roughness is a crucial property that needs to be tested in prosthodontics. It adds to material biocompatibility, antimicrobial adhesion and fatigue resistance [21, 49, 55]. Surface roughness was influenced by adding nanofillers, whicsignificantly increased the summary pooled value of the meta-analysis (*p* = 0.024). However, incorporating fillers as low as (0.1%) might be acceptable, as confirmed by the insignificant p-value of this subgroup (*p* = 0.182).

Different databases like PubMed, Web of Science, Cochrane Library and Wiley. While Scopus was not selected, the comprehensive coverage of biomedical literature through PubMed and Web of Science, coupled with citation snowballing, minimized the risk of missing any relevant studies.

The meta-analysis has attempted to reduce heterogeneity detected among the collected articles in various outcomes. Subgroup analysis was considered, relying on nanofiller concentration whenever possible. While the subgroup analyses by concentration reduced heterogeneity, variability in material fabrication methods (e.g., 3D printing vs. CAD/CAM) and testing protocols (e.g., different loading rates for flexural strength) introduced residual heterogeneity. These findings should be interpreted as preliminary, dealing with the conclusion cautiously and highlighting the need for standardized methodologies in future studies. Moreover, publication bias and sensitivity analysis were assessed when a valid number of articles existed. Sensitivity analysis was checked using the “one-study removed” method. It could be considered a strength point where no significant effect was recorded by removing specific studies. In addition, a positive point was seen while testing the flexural strength publication bias, as no publication bias was reported, as confirmed by the funnel plot and Egger’s test. On the other hand, the funnel plot asymmetry for surface roughness suggests potential selective reporting of positive outcomes. This bias may overestimate graphene’s efficacy in reducing roughness, necessitating cautious interpretation. This publication bias, particularly in surface roughness studies, may skew the literature toward favorable outcomes. This could arise from underreporting of null results, especially in industry-funded research. Independent replication studies are critical to reduce this risk.

Based on their study designs, the quality of the studies was assessed. It showed a variation in the risk of bias from low to high risk, which could be reflected in the heterogeneity level and publication bias. Only flexural strength and roughness were tested for publication bias, which is considered a limitation of this study. The number of studies was also limited and insufficient to reveal solid evidence for some properties, such as compressive and impact strength. It is also recommended that testing techniques and nanofillers’ incremental concentrations be standardized to minimize heterogeneity and the risk of bias.

Another limitation of this study was the lack of clinical studies, observational or experimental, especially when we see many studies testing graphene in vivo as a dental implant coating [68]. Randomized controlled trials (RCTs) are known as the gold standard in dentistry, and even if a higher nanofillers’ concentration is accepted mechanically, it may have biocompatibility and esthetic issues. In this case, a compromise between several properties might be needed [31]. Consequently, it is recommended that more in-depth biocompatibility studies be conducted and extended to the clinical level [11]. 

Although some mechanical properties were studied, key properties such as wear resistance and elastic modulus were underreported in the included studies, limiting our ability to assess graphene’s full impact on prosthodontic material performance.

Although a broad scope was presented in this review, excluding studies that use a hybrid method of mixing nanofillers may affect the number of articles collected. The recent trend of merging different nano-fillers has been popular in reducing the adverse effects or augmenting the dominant filler value [5, 7, 12, 53]. 

## Conclusion

Within the quality and heterogeneity level of the included studies, some conclusions could be drawn from the current systematic review and meta-analysis. Graphene and its derivatives, at specific concentrations, are promising nanofillers that can optimize the properties of the nanocomposites used for prosthodontic patients. Maximizing nanofiller concentration may have adverse effects on the material properties. While low-concentration graphene shows promise, high heterogeneity and methodological variability across studies preclude definitive clinical recommendations. Future research must prioritize standardized protocols and clinical validation. Sufficient clinical and biocompatibility studies are required to support the clinical implication of graphene usage on a broad spectrum. Advanced nanofillers’ dispersion in the polymer’s matrix should be considered to guarantee adequate distribution.

## Data Availability

No datasets were generated or analysed during the current study.

## Abbreviations

GO: Graphene oxide
rGO: Graphene oxide reduced form
GNPs: Graphene nano-platelets
MRI: Medical resonance image
Tg: Glass transition temperature
RCTs: Randomized controlled trials
NOS: Newcastle-Ottawa Scale for non-randomized studies
SMD: The standardized mean difference
E.S.: Effect size
S.E.: Standard errors
C.I.s: Confidence intervals
PMMA: Polymethyl Methacrylates
GNF: Graphene nano-fibers
nGO sheets: Nano-graphene oxide sheets
